# Design method for VDCC-based analog comb filter for power line interference cancellation

**DOI:** 10.1016/j.mex.2024.102619

**Published:** 2024-02-22

**Authors:** Chandan Kumar Choubey, Sumit Kumar, Sanjeev Kumar Pippal

**Affiliations:** a Symbiosis Institute of Technology, Pune Campus, Symbiosis International (Deemed University), Pune, India; b Department of Computer Science Engineering, Sharda School of Engineering and Technology, Sharda University, Greater Noida, Uttar Pradesh, India

**Keywords:** Notch filter, Comb filter, PLI, Biomedical signals, Methodology to design VDCC-based comb filter

## Abstract

An analog comb filter is implemented by linking multiple VDCC-based notch filters in a cascading fashion (N in total), eliminating N different pole frequencies. This study focuses on suppressing a fundamental frequency of power-line interference of 50 Hz and its consecutive three odd harmonics at 150 Hz, 250 Hz, and 350 Hz. One significant advantage of this comb filter is the independent control over filters' parameters like quality factor and pole frequency. Additionally, these filters can be electronically tuned by adjusting the transconductance gain of VDCC. The suggested notch filter configuration involves 2 capacitors, 2 resistors, and 1 VDCC element. Extensive simulations were conducted using PSPICE simulator software to validate the effectiveness of these filters. The basic building block, VDCC, is designed and implemented in the simulation using integrated circuits MAX435 and AD844.

• Design uses a VDCC-based high Q notch filter as the active building block.
• The filter employs fewer active and passive components.
• Simulated results using commercially available ICs, MAX435 and AD844, confirm the filter's practical utility.

## Graphical abstract

**Figure:**
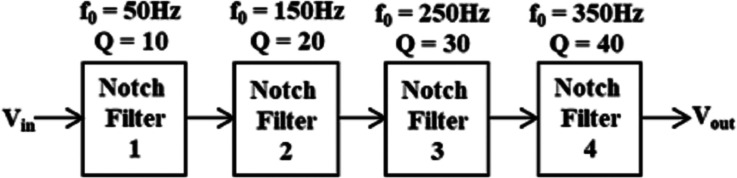

Specifications table

**Table:** 

Subject area:	*Engineering*
More specific subject area:	*Electronics circuit design*
Name of your method:	*Methodology to design VDCC-based comb filter*
Name and reference of original method:	*C.D. Tsai, D. C. Chiou, Y. D. Lin, H.L. Chan, and C.P. Wu, “An active comb filter design for harmonic interference removal,” Journal of the Chinese Institute of Engineers, vol. 21, no. 5, pp. 605–610, 1998.*
Resource availability:	*Method validation using ICs MAX435 and AD844 in PSPICE software.*

## Method details

To effectively eliminate power-line interference from biological signals such as electrocardiogram (ECG), electroencephalogram (EEG), and electromyogram (EMG), suppressing the primary frequency and a few odd harmonics associated with it is essential [1,2]. A valuable solution for achieving this is the utilization of a comb filter. Unlike notch filters, comb filters can attenuate multiple frequencies simultaneously. Analog filters hold a distinct advantage for real-time signal processing compared to digital counterparts. Consequently, numerous analog comb filters have been listed in existing literature [3], [4], [5], [6], [7], [8], [9] to eliminate the primary frequency of PLI and its associated odd harmonics from biological signals like electrocardiogram (ECG), electroencephalogram (EEG), and electromyogram (EMG).

The key benefits of the suggested comb filter, which represents the novelty of the circuit by overcoming the literature research gaps, can be summarized as follows:

• Minimal Active Building Blocks: The proposed comb filter is notably efficient regarding active components, requiring only 4 VDCCs. Meanwhile, in [3], [4], [5], [6], [7], [8], more than four active building blocks are used.
• Reduced Passive Components: Besides its frugality with active components, the proposed filter employs fewer passive components than [3], [4], [5], [6], [7], [8].
• Low MOSFET Count: Compared to alternative approaches in [3], [4], [5], [6], [7], [8], the proposed filter demands a mere 48 MOSFETs, contributing to its efficiency and simplicity.
• Orthogonal Parameter Relationship: The filter establishes an orthogonal correlation between the pole frequency and the quality factor, enhancing flexibility and adaptability for various applications.

The proposed comb filter has only one limitation: the notch depth is lesser than the existing comb filters. Still, it is sufficient to attenuate the power-line interference effectively. Also, a high notch depth may distort the output signal.

**Fig. 1:**
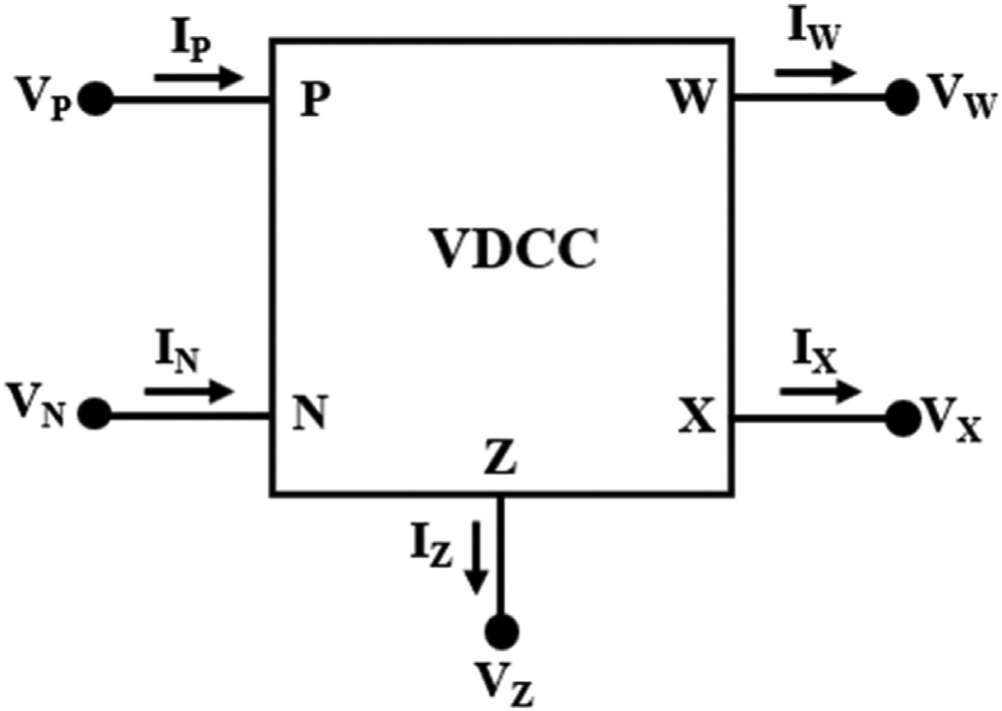
Electrical symbol of VDCC.

Due to the inherent properties of current mode circuits, including lower power consumption, wider bandwidth, higher dynamic range, and simpler architecture, several current mode blocks have gained prominence in designing various analog signal processing and generating circuits. These include second-generation current conveyor (CCII) [10], voltage differencing transconductance amplifier (VDTA) [11], differential difference current conveyor (DDCC) [12], voltage differencing gain amplifier (VDGA) [13], multiple output current differencing transconductance amplifier (MOCDTA) [14], and voltage differencing current conveyor (VDCC) [15,16]. The VDCC is particularly noteworthy for its ability to offer electronically adjustable transconductance gain and facilitate the concurrent current and voltage transfer across its terminals. This inherent versatility renders the VDCC highly conducive to the development of active filters and inductor simulators, among other applications. The VDCC, depicted in Fig. 1, is an analog building block featuring five terminals, P, N, X, Z, and W. P and N are high-impedance input terminals, Z and W are high-impedance output terminals, and X is low-impedance output terminal. The VDCC comprises an operational transconductance amplifier (OTA) with a transconductance gain represented as “g_m_,” followed by a second-generation current conveyor (CCII) in cascade mode. The relationships among the various terminals of the VDCC are described in [16] as given in Eq. (1).

(1) $$\left[\begin{array}{c} \begin{array}{c} I_{P}\\ I_{N}\\ I_{Z} \end{array}\\ V_{X}\\ I_{W} \end{array}\right]\;=\left[\begin{array}{c} \begin{array}{c} 0\\ 0\\ g_{m} \end{array}\\ 0\\ 0 \end{array}\begin{array}{c} \begin{array}{c} 0\\ 0\\ -g_{m} \end{array}\\ 0\\ 0 \end{array}\begin{array}{c} \begin{array}{c} 0\\ 0\\ 0 \end{array}\\ 1\\ 0 \end{array}\;\begin{array}{c} \begin{array}{c} 0\\ 0\\ 0 \end{array}\\ 0\\ 1 \end{array}\right]\left[\begin{array}{c} V_{P}\\ V_{N}\\ V_{Z}\\ I_{X} \end{array}\right]$$

**Fig. 2:**
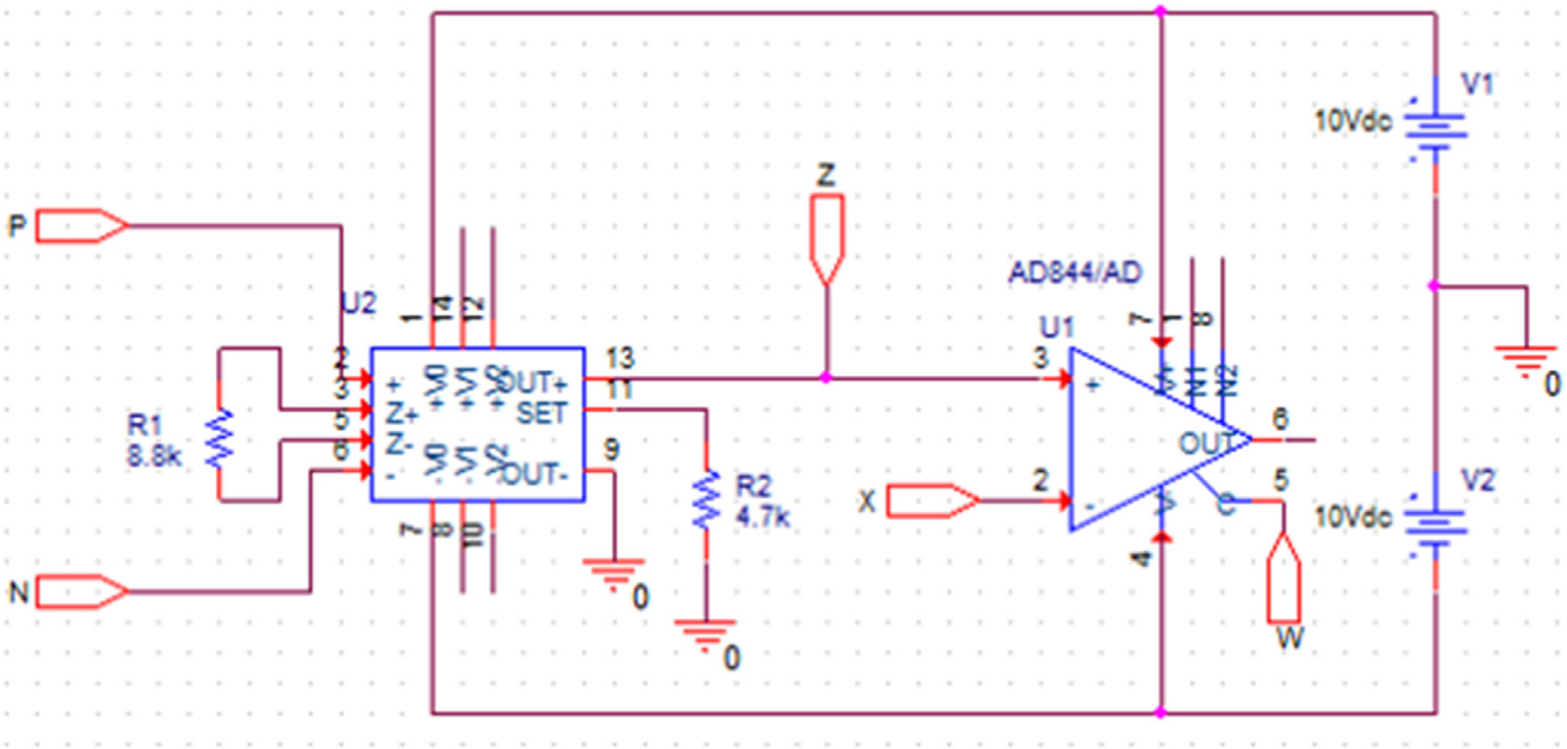
Implementation of VDCC using ICs MAX435 and AD844.

The port relationship in matrix form in Eq. (1) can be understood as follows. The VDCC operates as follows: The differential input voltage (*V_P_* - *V_N_*) undergoes multiplication by the transconductance gain (g_m_) of the VDCC, resulting in a current output, denoted as *I_Z_*, which is accessible at output terminal *Z*. Simultaneously, the voltage *V_Z_* is present at terminal *X*, and it is equivalent to *V_Z_*. The current at terminal *X* is then conveyed to terminal W, meaning I_W_ = I_X_. Notably, no current flows through the *P* and *N* terminals, leaving *I_P_* and *I_N_* equal to zero because of the high input impedance of the VDCC. An IC-based implementation of VDCC using an OTA IC MAX435 and a CFOA IC AD844, shown in Fig. 2, is used in this research article. The exact VDCC implementation will be employed for simulation and has been explored for hardware implementation.

**Fig. 3:**
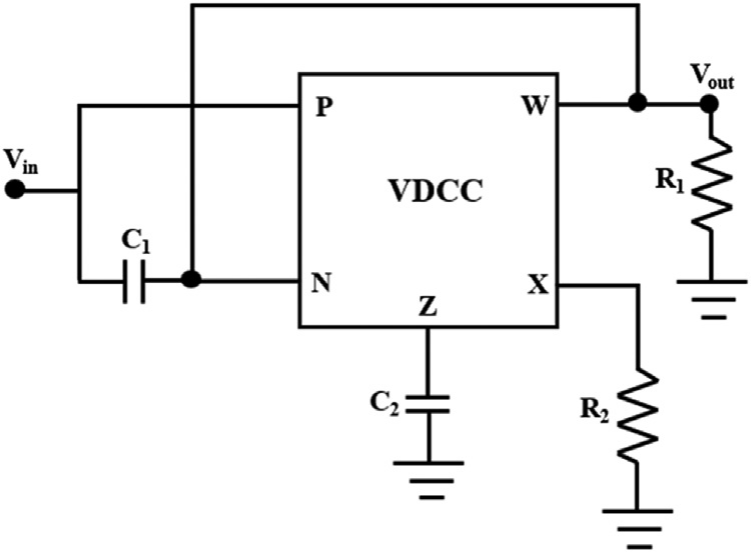
Circuit diagram of a single VDCC-based notch filter.

A single VDCC-based notch filter has been implemented, as presented in Fig. 3. In this implementation, 2 capacitors and 2 resistors are employed as the passive components. A VDCC is used as an active block in this filter.

The transfer function is obtained by standard analysis of Fig. 3 as follows.

(2) $$H\left(s\right)=\frac{V_{out}\left(s\right)}{\;V_{in}\left(s\right)}=\frac{s^{2}+\;\frac{g_{m}}{R_{2}C_{1}C_{2}}\;}{s^{2}+\frac{\;s}{R_{1}C_{1}}+\;\frac{g_{m}}{R_{2}C_{1}C_{2}}\;}$$

The second-order notch filter transfer function can be expressed as:

(3) $$H\left(s\right)=\frac{s^{2}+\omega_{0}^{2}}{s^{2}+s\left(\frac{\omega_{0}}{Q}\right)+\omega_{0}^{2}\;}$$

By comparing the coefficients in Eqs. (2) and (3), we can derive the values of the pole frequency (*ω*_o_) in rad/sec and the quality factor (*Q*) for the notch filter as follows:

(4) $$f_{0}=\frac{1}{2\pi }\sqrt{\frac{g_{m}}{R_{2}C_{1}C_{2}}}$$

(5) $$Q=R_{1}\sqrt{\frac{g_{m}C_{1}}{R_{2}C_{2}}}$$

Observing Eqs. (4) and (5), it becomes evident that the pole-frequency and the quality factor can be individually tuned to achieve specific values, allowing for independent control of these crucial parameters. In this design, we have intentionally set the value of *R*_2_ as *R*_2_= 1/*g_m_* for ease in setting the components’ values for various pole frequencies and quality factors. Considering *R*_2_= 1/*g_m_*, (2), (4), and (5) can be rewritten as:

(6) $$H\left(s\right)=\frac{V_{out}\left(s\right)}{\;V_{in}\left(s\right)}=\frac{s^{2}+\;\frac{gm^{2}}{C_{1}C_{2}}\;}{s^{2}+\frac{\;s}{R_{1}C_{1}}+\;\frac{gm^{2}}{C_{1}C_{2}}\;}$$

(7) $$f_{0}=\frac{g_{m}}{2\pi }\sqrt{\frac{1}{C_{1}C_{2}}}$$

(8) $$Q=g_{m}R_{1}\sqrt{\frac{C_{1}}{C_{2}}}$$

The manipulation of *g_m_, C*_1_, and *C*_2_ provides the means to fine-tune the pole frequency to the desired value. In contrast, by employing *R*_1_, the quality factor can be independently adjusted while keeping gm, *C*_1_, and *C*_2_ constant, as depicted in Eqs. (7) and (8). The same notch filter is used in the proposed comb filter, discussed in the next section.

**Fig. 4:**
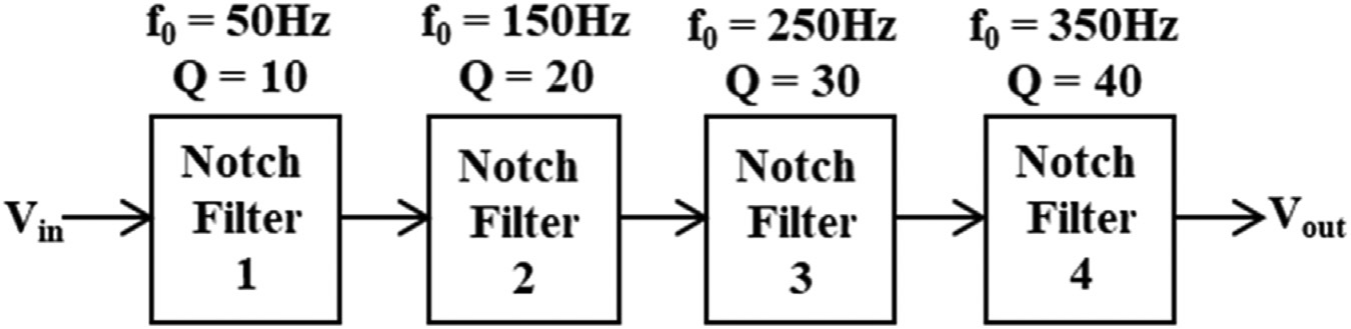
Methodology to design analog comb filter.

A novel analog comb filter using VDCC is discussed here. The filter, which can sharply attenuate more than one frequency, is called a comb filter. Its name is given due to the comb-like magnitude response in the frequency domain. One of the methods to synthesize a comb filter is by connecting N number of notch filters for suppressing N number of pole frequencies. We have chosen four notch filters in the proposed design for suppressing four pole frequencies, one PLI, and its three odd harmonics. The cascading of four notch filters for four different pole frequencies with four different quality factors is presented in Fig. 4. The notch filters are precisely tuned to target specific frequencies, including the fundamental frequency of power line interference at 50 Hz, as well as the 3^rd^, 5^th^, and 7^th^ odd harmonics at 150 Hz, 250 Hz, and 350 Hz. 10, 20, 30, and 40 quality factors have been thoughtfully selected to achieve a sharp and effective notching performance.

**Fig. 5:**
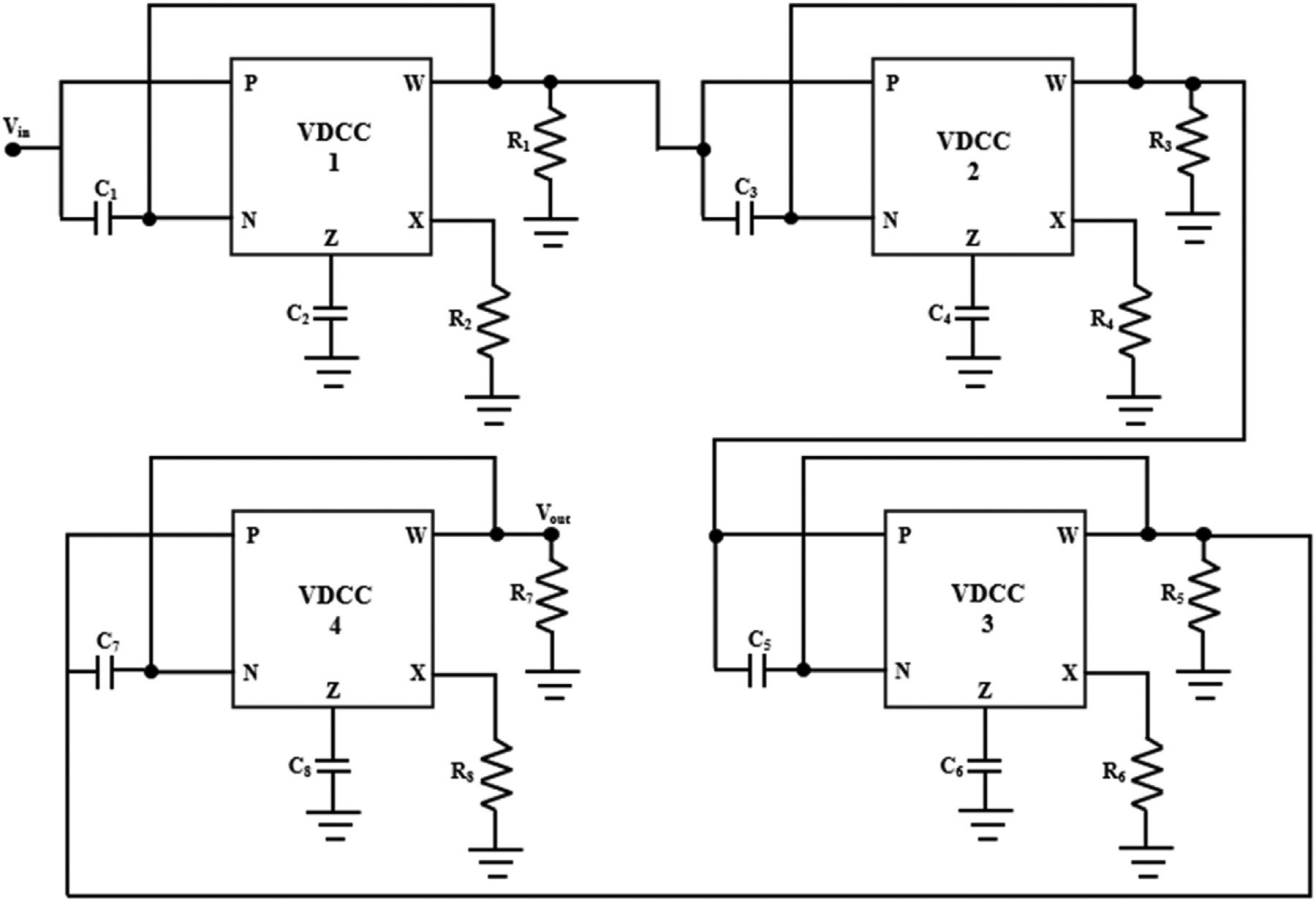
Comb filter using VDCC-based notch filters for n= 4.

The VDCC comb filter circuit, which is built upon the VDCC-based approach and incorporates four notch filters, is visually presented in Fig. 5. This design utilizes 4 Voltage Differencing Current Conveyors (VDCCs), 8 capacitors, and 4 resistors to achieve its functionality. The transconductance gain of all four VDCCs is taken the same as g_m_ because the same VDCC has been used for all the four notch filters. In the proposed comb filter, eight capacitors, *C*_1_, *C*_2_, *C*_3_, *C*_4_, *C*_5_, *C*_6_, *C*_7_, and *C*_8_, and four resistors, *R*_1_, *R*_2_, *R*_3_, and *R*_4_ are used. By multiplying the transfer functions of four successively linked notch filters, it is possible to get the transfer function of the suggested comb filter shown in Fig. 5.

(9) $$H\left(s\right)=\frac{s^{2}+\;\frac{g_{m1}}{R_{2}C_{1}C_{2}}\;}{s^{2}+\frac{\;s}{R_{1}C_{1}}+\;\frac{g_{m1}}{R_{2}C_{1}C_{2}}\;}\times \frac{s^{2}+\;\frac{g_{m2}}{R_{4}C_{3}C_{4}}\;}{s^{2}+\frac{\;s}{R_{3}C_{3}}+\;\frac{g_{m2}}{R_{4}C_{3}C_{4}}\;}\times \frac{s^{2}+\;\frac{g_{m3}}{R_{6}C_{5}C_{6}}\;}{s^{2}+\frac{\;s}{R_{5}C_{5}}+\;\frac{g_{m3}}{R_{6}C_{5}C_{6}}\;}\times \frac{s^{2}+\;\frac{g_{m4}}{R_{8}C_{7}C_{8}}\;}{s^{2}+\frac{\;s}{R_{7}C_{7}}+\;\frac{g_{m4}}{R_{8}C_{7}C_{8}}\;}$$

For *R*_2_= *R*_4_= *R*_6_= *R*_8_= 1/*g_m_*, (9) gives:

(10) $$H\left(s\right)=\frac{s^{2}+\;\frac{gm^{2}}{C_{1}C_{2}}\;}{s^{2}+\frac{\;s}{R_{1}C_{1}}+\;\frac{gm^{2}}{C_{1}C_{2}}\;}\times \frac{s^{2}+\;\frac{gm^{2}}{C_{3}C_{4}}\;}{s^{2}+\frac{\;s}{R_{3}C_{3}}+\;\frac{gm^{2}}{C_{3}C_{4}}\;}\times \frac{s^{2}+\;\frac{gm^{2}}{C_{5}C_{6}}\;}{s^{2}+\frac{\;s}{R_{5}C_{5}}+\;\frac{gm^{2}}{C_{5}C_{6}}\;}\times \frac{s^{2}+\;\frac{gm^{2}}{C_{7}C_{8}}\;}{s^{2}+\frac{\;s}{R_{7}C_{7}}+\;\frac{gm^{2}}{C_{7}C_{8}}\;}$$

The filter's parameters can be expressed in Eqs. (11) and (12) as:

(11) $$Pole-frequencies\;\;:\;f_{01}=\frac{g_{m}}{2\pi }\sqrt{\frac{1}{C_{1}C_{2}}};\;f_{02}=\frac{g_{m}}{2\pi }\sqrt{\frac{1}{C_{3}C_{4}}};\;f_{03}=\frac{g_{m}}{2\pi }\sqrt{\frac{1}{C_{5}C_{6}}};\;f_{04}=\frac{g_{m}}{2\pi }\sqrt{\frac{1}{C_{7}C_{8}}}$$

(12) $$Quality\;factors\;\;:\;Q_{1}=g_{m}R_{1}\sqrt{\frac{C_{1}}{C_{2}}};\;Q_{2}=g_{m}R_{3}\sqrt{\frac{C_{3}}{C_{4}}};\;Q_{3}=g_{m}R_{5}\sqrt{\frac{C_{5}}{C_{6}}};\;Q_{4}=g_{m}R_{7}\sqrt{\frac{C_{7}}{C_{8}}}$$

Much like the parameters of the notch filter, these comb filter's parameters can be independently tuned to cater to specific requirements.

## Validation

The suggested notch and comb filters of Figs. 3 and 5 are simulated using PSPICE. The analog building block of these filters, VDCC, is implemented using two high-performance ICs, MAX435 and AD844, in the simulation and the hardware implementation. IC MAX435 and IC AD844 are OTA (Operational Transconductance Amplifier) and CFOA (Current Feedback Operational Amplifier) ICs, respectively. These two ICs are cascaded to implement a versatile current-mode building block, VDCC. The simulation setup of the IC-based VDCC implementation is shown in Fig. 2. This setup is used for the simulation and hardware implementation of the proposed notch and comb filter. For the proper biasing of these ICs, power supplies are taken as *V_DD_*= +10V and *V_SS_*= -10 V. The transconductance gain, *gm*, of the VDCC is set by resistor *R*_1_ of Fig. 2 using a predefined relation between g_m_ and limiting resistor *R*1 as *g_m_*= 4/*R*_1_. From this relation, *gm* can be calculated as 454.54 µA/V. This *g_m_* value has been used for the simulation and hardware implementation of VDCC-based filters. The resistor *R*_2_ used in Fig. 2 is the set resistor of IC MAX435. The value of *R*_2_ is carefully chosen as 4.7 kΩ. The terminals of VDCC, P, N, Z, X, and W, are made as the input-output ports and have been used in the VDCC block. The same block has been used to simulate notch and comb filters, shown in Fig. 3 and Fig. 5, respectively.

**Fig. 6:**
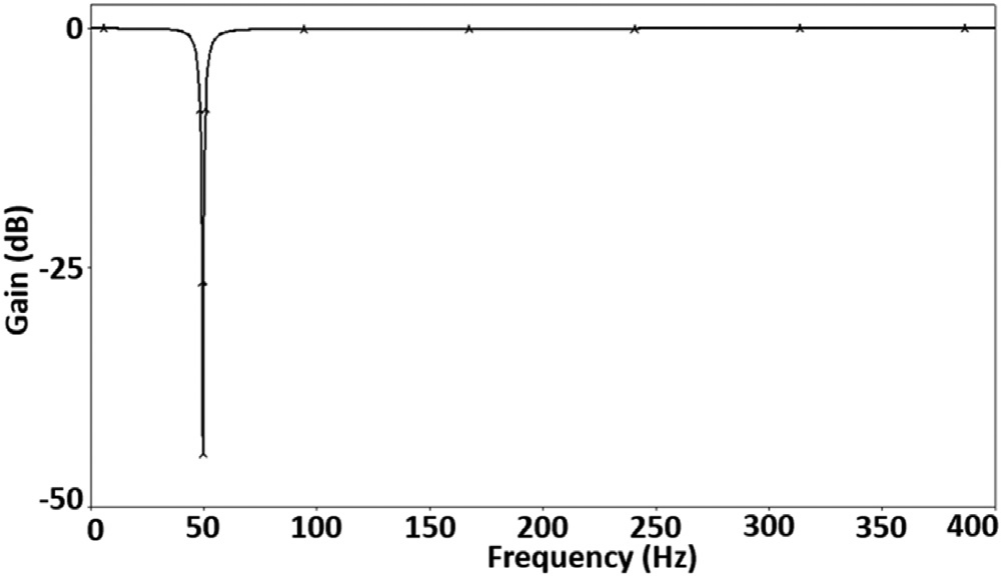
Magnitude frequency response of the notch filter.

**Fig. 7:**
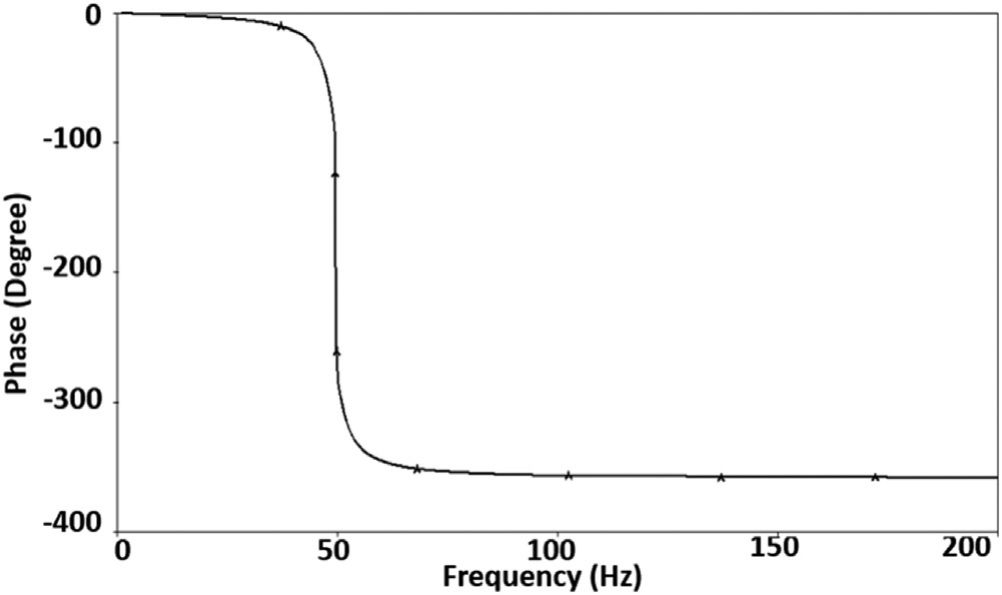
Phase frequency response of the notch filter.

First, the notch filter has been designed for a pole frequency of 50Hz and a quality factor of 10 to suppress the 50Hz PLI effectively. For this design, the passive components (capacitors and resistors) used in Fig. 3 are chosen as *C*_1_= 362 nF, *C*_2_= 5.8 µF, *R*_1_= 5.5 kΩ, and *R*_2_= 2.2 kΩ. As discussed above, the transconductance gain of VDCC, *gm*, is set to a value of 454.54 µA/V. In Fig. 6, Fig. 7, you can observe the notch filter's simulated magnitude and phase responses. These responses vividly demonstrate the remarkable efficacy of the proposed notch filter in attenuating the fifty-hertz power line interference with a high degree of precision and sharp notching. In magnitude response, shown in Fig. 6, a notch depth of -45.7dB is obtained, effectively suppressing the 50Hz PLI. Phase response, shown in Fig. 7, shows a sharp phase change of 360⁰ from 0⁰ to -360⁰ at the pole frequency.

**Fig. 8:**
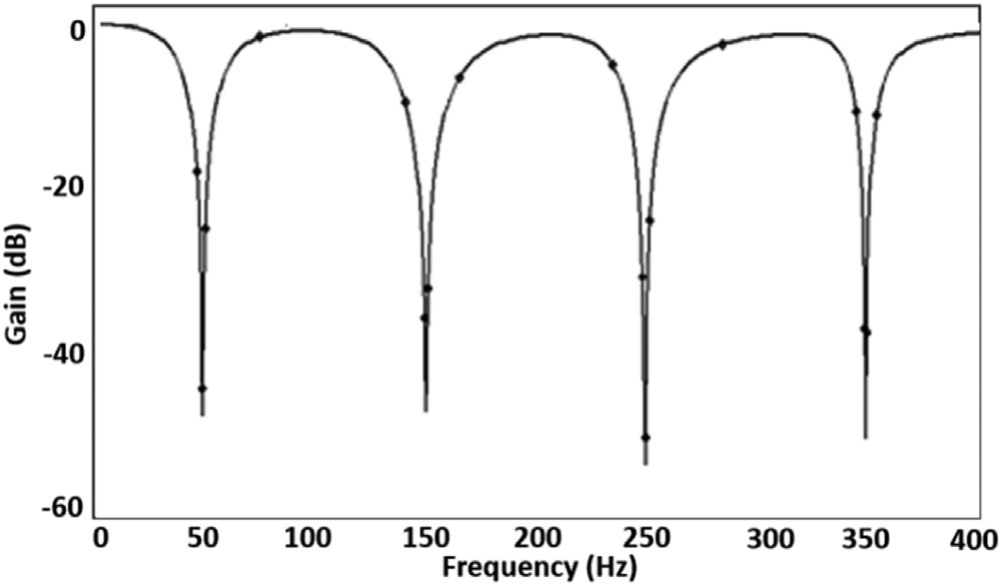
Magnitude frequency response of the comb filter.

The proposed comb filter, shown in Fig. 5, has been simulated with the same setup of VDCC. The values of passive components (capacitors and resistors) used in Fig. 5 are given in Table 1. With these values of capacitors and resistors, the four cascaded notch filters are set for the pole frequency of 50 Hz, 250 Hz, and 350 Hz and quality factors of 10, 20, 30, and 40, respectively. The magnitude response corresponding to these settings is visually depicted in Fig. 8.

**Table 1:** Values of passive components.

*Sr. No.*	*Passive Component Values*	*Pole frequency (f_0_)*	*Quality Factor (Q_0_)*
*1*	*C_1_ = 362 nF, C_2_ = 5.8 µF, R_1_ = 5.5 kΩ, R_2_ = 2.2kΩ*	*50Hz*	*10*
*2*	*C_3_ = 96.46 nF, C_4_ = 2.4 µF, R_3_ = 8.8 kΩ, R_4_ = 2.2kΩ*	*150Hz*	*20*
*3*	*C_5_ = 48.23 nF, C_6_ = 1.74 µF, R_5_ = 11 kΩ, R_6_ = 2.2kΩ*	*250Hz*	*30*
*4*	*C_7_ = 29.53nF, C_8__=_ 1.45µF, R_7_ = 12.54 kΩ, R_8_ = 2.2 kΩ*	*350Hz*	*40*

**Fig. 9:**
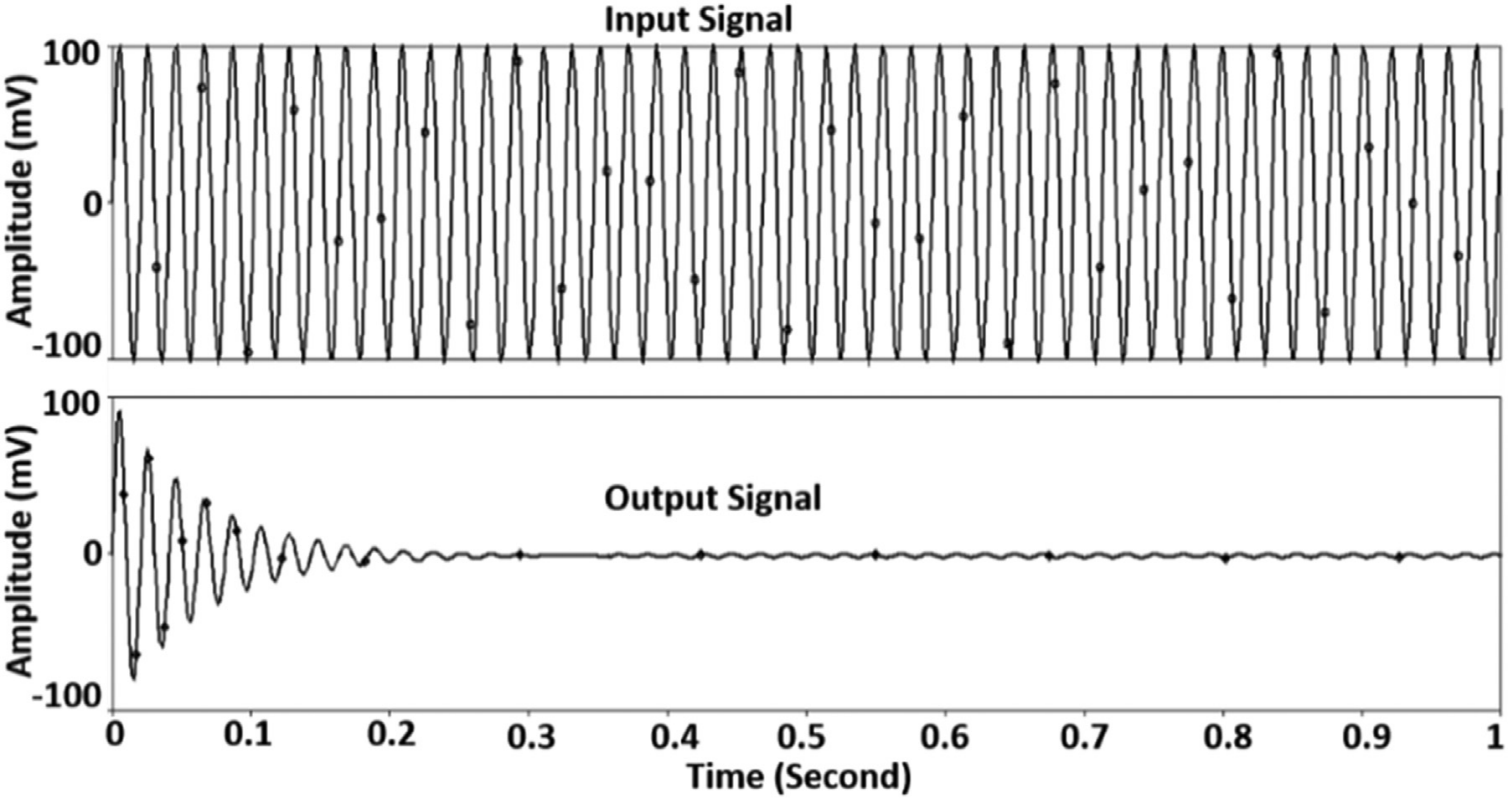
Time domain response of the comb filter.

A sinusoidal waveform is run through the suggested comb filter to assess its effectiveness in the time domain. The frequency and the amplitude are taken as 50Hz and 100mV, respectively. The 50Hz waveform resembles the 50Hz PLI, whereas 100mV amplitude is sufficient to resemble the amplitude of PLI in low-frequency, low-amplitude biological signals. The input and output waveforms are shown in Fig. 9. It can be seen that the 50Hz input signal is well suppressed after some settling time, as expected. It verifies in real-time how well the suggested comb filter performs.

## Conclusion

A VDCC-based analog comb filter is designed to suppress unwanted power-line interference from biomedical signals like electrocardiogram (ECG), electroencephalogram (EEG), and electromyogram (EMG). The filter is designed by cascading four notch filters of pole frequencies set at 50 Hz, 150 Hz, 250 Hz, and 350 Hz, each having quality factors of 10, 20, 30, and 40, respectively. By setting these pole frequencies and quality factors, it is observed in the simulation that the filter effectively suppresses the 50Hz power-line interference and its three consecutive odd harmonics of 150 Hz, 250 Hz, and 350 Hz. The filter is simulated using macro-models of integrated circuits MAX435 and AD844. The filter circuit uses fewer active and passive components. Also, this filter's architecture can achieve the high-quality factor for the sharp notching of the 50 Hz power-line interference (Table 2).

**Table 2:** Comparision of VDCC and Non-VDCC comb filter.

*Ref. No.*	*Active block*	*Number of Active Component*	*Number of Passive Components*
*3*	*Op-Amp*	*OpAmp = 5*	*Resistor = 18* *Capacitor = 8*
*4*	*Op-Amp*	*OpAmp = 5*	*Resistor = 18* *Capacitor = 8*
*5*	*OTA*	*OTA = 10*	*Capacitor = 8*
*6*	*CCII*	*CCII = 9*	*Resistor = 19* *Capacitor = 8*
*7*	*CCII*	*OpAmp = 11*	*Resistor = 11* *Capacitor = 8*
*8*	*CCII*	*OpAmp = 17*	*Resistor = 17* *Capacitor = 8*
*9*	*VDCC*	*VDCC = 4*	*Resistor = 8* *Capacitor = 8*

## Future scope

The filter's active building block, VDCC, can be implemented with the latest CMOS technology parameters like 90nm, 45nm, and 22nm for fabricating the circuit in IC form. Further, there may be scope for reducing the number of passive and active components by introducing a new active building block.

## Ethics statements

This research did not involve research on humans or animals, and no data is involved from social media platforms.

## CRediT authorship contribution statement

**Chandan Kumar Choubey:** Conceptualization, Methodology, Visualization, Validation, Writing – review & editing, Writing – original draft. **Sumit Kumar:** Validation, Data curation, Supervision. **Sanjeev Kumar Pippal:** Visualization, Investigation.

## Declaration of competing interest

The authors declare that they have no known competing financial interests or personal relationships that could have appeared to influence the work reported in this paper.

## Data Availability

No data was used for the research described in the article.

## References

[1] Singh B., Singh P., Budhiraja S. (2015). Proceedings of the Fifth International Conference on Advanced Computing & Communication Technologies.

[2] Ziarani A.K., Konrad A.A. (2002). Non-linear adaptive method of elimination of power line interference in ECG signals. IEEE Trans. Biomed. Eng..

[3] Tsai C.D., Chiou D.C., Lin Y.D., Chan H.L., Wu C.P. (1998). An active comb filter design for harmonic interference removal. J. Chin. Inst. Eng..

[4] Tsai C.T., Chan H.L., Tseng C.C., Wu C.P. (1994). Proceedings of 16th Annual International Conference of the IEEE Engineering in Medicine and Biology Society.

[5] Ranjan R.K., Yalla S.P., Sorya S., Paul S.K. (2014). Active comb filter using operational transconductance amplifier. Act. Pass. Electr. Comp..

[6] Ranjan R.K., Choubey C.K., Nagar B.C., Paul S.K. (2016). Comb filter for elimination of unwanted power line interference in biomedical signal. J. Circ. Syst. Comput..

[7] Paul S.K., Choubey C.K., Tiwari G. (2018). Low power analog comb filter for biomedical applications. Analog. Integr. Circuits. Signal. Process..

[8] Choubey C.K., Sahani A., Paul S.K. (2016). Proceedings of the IEEE International Conference on Advances in Electronics, Communication and Computer Technology (ICAECCT).

[9] Choubey C.K., Kanungo A., Srivastava A., Gupta A. (2023). Proceedings of the 3rd Asian Conference on Innovation in Technology (ASIANCON).

[10] Choubey C.K., Tiwari G., Paul S.K. (2016). Proceedings of the IEEE International Conference on Advances in Electronics, Communication and Computer Technology (ICAECCT).

[11] Choubey C.K., Paul S.K. (2020). Implementation of chaotic oscillator by designing a simple Chua's diode using a single VDTA. AEU - Int. J. Electr. Commun..

[12] Choubey C.K., Paul S.K. (2022). Nth order voltage-mode universal filter employing only plus type differential difference current conveyor. Analog Integr. Circ. Sig. Process..

[13] Choubey C.K., Paul S.K. (2023). Systematic realisation of inductorless and resistorless Chua's chaotic oscillator using VDGA. Int. J. Electr..

[14] Kumar A., Kumar S., Elkamchouchi D.H., Urooj S. (2022). Fully differential current-mode configuration for the realization of first-order filters with ease of cascadability. Electronics. (Basel).

[15] Kaçar F., Yeşil A., Minaei S., Kuntman H. (2014). Positive/negative lossy/lossless grounded inductance simulators employing single VDCC and only two passive elements. AEU - Int. J. Electr. Commun..

[16] Sotner R., Jerabek J., Petrzela J., Dostal T. (2016). Proceedings of the International Conference on Applied Electronics (AE).

